# Diallyl trisulfide inhibits osteosarcoma 143B cell migration, invasion and EMT by inducing autophagy

**DOI:** 10.1016/j.heliyon.2024.e26681

**Published:** 2024-02-22

**Authors:** Xiyu Liu, Nan Wang, Zhiwei He, Chen Chen, Jun Ma, Xin Liu, Shan Deng, Lin Xie

**Affiliations:** a The Third Clinical Medical College, Nanjing University of Chinese Medicine, Nanjing, China; b Yancheng TCM Hospital Affiliated to Nanjing University of Chinese Medicine, Yancheng City, China; c Huai’an TCM Hospital Affiliated to Nanjing University of Chinese Medicine, Huai’an, China

**Keywords:** Diallyl trisulfide, Osteosarcoma, Autophagy, EGFR/PI3K/AKT/mTOR

## Abstract

**Background:**

Diallyl trisulfide (DATS), a compound derived from garlic, has been demonstrated its anti-cancer properties. While it has been shown to inhibit the expression of epidermal growth factor receptor (EGFR) in various cancers, its effects on osteosarcoma (OS) cells remain unclear. This study aimed to investigate the impacts of DATS on OS cells growth, migration, invasion, epithelial-mesenchymal transition (EMT) and autophagy, as well as its underlying mechanisms which was involving in the EGFR/PI3K/AKT/mTOR pathway.

**Methods:**

In this study, human osteosarcoma cells (143B) were treated with different concentrations of DATS (10, 50, 100 and 200 μM) for 24 and 48 h, respectively. Cell viability was measured using CCK8, the half lethal concentration was selected for the following experiments. Wound healing and transwell assays were performed to evaluate migration and invasion abilities, while flow cytometry was used to measure apoptosis. Quantitative reverse transcription polymerase chain reaction (qRT-PCR), Western blotting, and confocal imaging were employed to analyze the related mRNA and protein expression levels of epithelial-mesenchymal transition (EMT), EGFR/Phosphoinositide 3 kinase (PI3K)/AKT/Mammalian target of rapamycin (mTOR) signaling pathway and autophagy-related markers.

**Results:**

DATS significantly inhibited proliferation, migration and EMT in osteosarcoma cells. Additionally, DATS promoted cell apoptosis and induced autophagy, which could be rescued by the autophagy inhibitor 3-methyladenine (3-MA). Moreover, DATS treatment led to the inactivation of the EGFR/PI3K/AKT/mTOR pathway in osteosarcoma cells.

**Conclusions:**

This study demonstrated that DATS inhibited osteosarcoma cell growth, migration and EMT, but inducing apoptosis and autophagy. These effects were mediated by the inactivation of the EGFR/PI3K/AKT/mTOR signaling pathway. These findings suggested that DATS could serve as a potential therapeutic agent for osteosarcoma treatment.

## 1 Introduction

Osteosarcoma (OS) is the most prevalent bone malignancy characterized by locally aggressive growth and early metastatic potential in children and adolescents. The incidence rate of OS is 2 cases per million, with a 5-year survival rate of less than 68% under 14 years old in the United States [1]. Despite significant advances in the treatment of OS, including surgical resection and the use of chemotherapy drugs such as cisplatin, methotrexate, and doxorubicin [[2], [3], [4]], the subsequent side effects, for example myelosuppression, nephrotoxicity, cardiac issues, and neurotoxicity, profoundly reduces patients’ quality of life [5,6]. Therefore, the novel effective and non-toxic agents are urgently needed to improve the therapeutic efficacy in osteosarcoma.

Autophagy plays a crucial role in assessing the efficacy of anticancer drugs, as it facilitates the transport of cytoplasmic substances to lysosomes for degradation through vesicles. It is the primary mechanism by which eukaryotes degrade their own organelles or proteins to regulate cell proliferation and functions [7,8]. Growing evidence indicated that autophagy has a significant role in tumor progression. By modulating various autophagy-specific markers, like MAP1LC3 (LC3) and Beclin-1, autophagy can control tumor growth [9]. The phosphatidylinositol 3-kinase (PI3K) family of enzymes are involved in several cellular functions, including cell growth, proliferation, movement, differentiation and intracellular transport. Protein kinase B (AKT) and mammalian rapamycin target (mTOR) are crucial regulators of autophagy in tumors. Recent studies have demonstrated that the PI3K/AKT/mTOR pathway is implicated in autophagy arrest in osteosarcoma cells [10]. Moreover, the epidermal growth factor receptor (EGFR) has emerged as an autophagy marker in OS [11]. Also the increased EGFR can rapid the malignant process of osteosarcoma [12]. Consequently, EGFR/AKT/mTOR inhibitors have attracted considerable attention in cancer research.

Diallyl trisulfide (DATS), also known as allicin, is the principal bioactive component found in garlic. It has been extensively studied and proven to possess anti-inflammatory, antiseptic, cardiovascular protective, and immune-enhancing properties [13]. Importantly, numerous studies have reported the inhibitory effects of DATS in various cancers, including breast cancer, gastric cancer, glioblastoma, hepatocellular carcinoma, esophageal cancer, and bladder cancer [[14], [15], [16], [17], [18]]. Furthermore, it has been reported that DATS has the ability to reduce the risk of gastrointestinal cancers [19], reverse chemotherapy resistance [20,21], and enhance anti-cancer activity when combined with biomaterials [22]. These findings highlight that DATS has potential to fight against cancer due to its multifaceted and multi-targeted feature, as well as its apparent lack of toxicity [23]. In recent years, although some progress has been made in exploring the molecular functions of DATS in OS [[24], [25], [26]], the underlying mechanism remains unclear.

Baesd on above, we aim to found out how DATS regulates osteosarcoma cell malignant processes. Our investigation revealed that DATS could inhibit proliferation, migration and EMT, but induce autophagy and apoptosis in OS cells by downregulating EGFR, and its downstream PI3K/AKT/mTOR signaling pathway. These results confirmed that DATS might be a useful candidates for OS therapy.

## 2 Results

### 2.1 DATS inhibited OS cells growth, migration and EMT

**Fig. 1:**
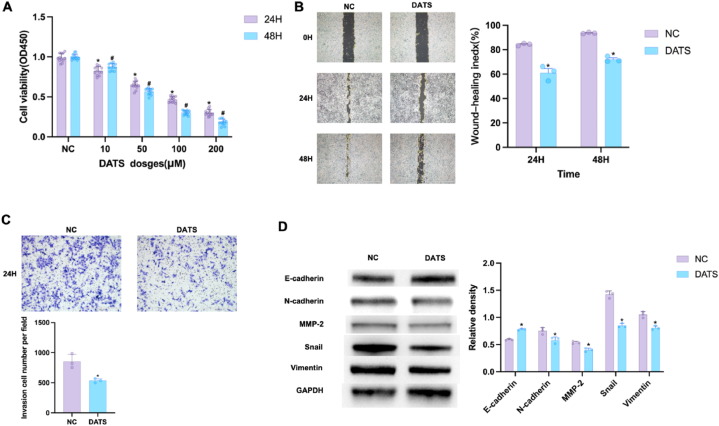
DATS inhibited OS cells growth, migration, invasion and EMT. (A) OS cells were treated with DATS (0, 10, 50, 100, 200 μM) for 24 and 48 h, CCK8 assay was conducted to test cell viability of OS cells. (B–C) Wound healing and transwell were used to assess the potential of DATS on migrative and invasive abilities. Scale bar: 1 mm for B and 500 μm for C. (D) Western blot was employed to measure the expression levels of E-cad, N-cad, MMP-2, Snail and Vimentin in OS cells. *p < 0.05 (compared with NC). #*p* < 0.05 (compared with NC). Data are represented as means ± SD; n = 3 per group.

First, OS cells viability was determined by CCK8 assay. As shown in Fig. 1A, the cell viabilities were significantly reduced in response to 24 and 48 h DATS treatment in a dose-dependent manner. DATS induced approximately 50% growth inhibition in OS cells after 24 h incubation. Therefore, 100 μM of DATS was used as working condition in the following experiments. To assess the potential of DATS in migration and invasion, we conducted wound healing and transwell experiments. After 48 h, the scratch gap area in the DATS group decreased slowly compared with the normal control (NC) group, as shown in Fig. 1B. The transwell results showed a significant decrease in the number of invasive cells, upon DATS treatment for 24 h (Fig. 1C), indicating that DATS blocked the migratory and invasive abilities of OS cells. To further investigate the effect of DATS on epithelial-mesenchymal transition (EMT), five EMT-related proteins—E-cadherin (E-cad), N-cadherin (N-cad), matrix metalloproteinase-2 (MMP-2), vimentin, and the E-cad blocker snail—were examined using Western blot analysis. The results showed that DATS suppressed the protein levels of N-cad, Snail, MMP-2 and Vimentin, but increased the level of E-cad in OS cells (Fig. 1D). These findings indicated that DATS inhibited cell growth, migration, invasion and EMT in OS.

### 2.2 DATS promoted OS cells apoptosis and autophagy

**Fig. 2:**
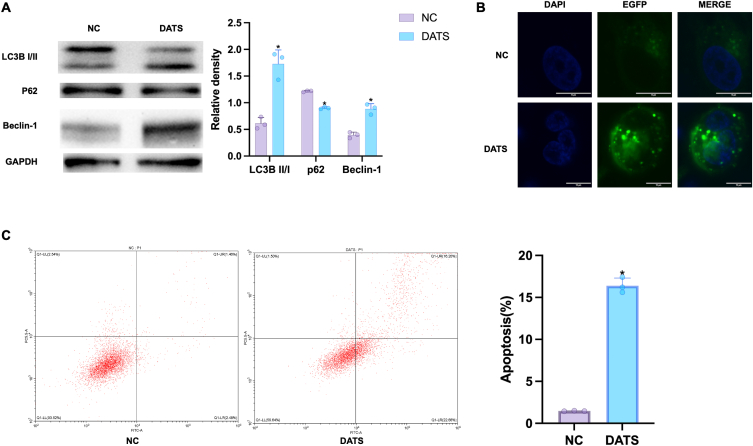
DATS induced autophagy and apoptosis. (A) Western blot was employed to measure the expression of LC3BI/II, p62 and Beclin-1 in OS cells after DATS treatment. (B) OS cells were transfected with a pEGFP-C3-MAP1LC3B reporter (green) and stained with DAPI (blue), then analyzed with confocal microscopy, scale bar = 10 μm. (C) The apoptosis rates of OS cells were examined by flow cytometry after the treatment of DATS for 24 h. *p < 0.05 (compared with NC). (For interpretation of the references to colour in this figure legend, the reader is referred to the Web version of this article.)

To estimate the effect of DATS on autophagy, Western blot was carried out to examine the autophagic markers p62, LC3B I/II, and Beclin-1. LC3B II is an indicator of autophagic induction, formed by the conversion of cytoplasmic LC3B I [27]. Thus, in Fig. 2A, we observed an significantly increase in the conversion rate from LC3B–I to LC3B-II in DATS group, along with the upregulation of Beclin-1, an autophagy promoter. The expression of autophagy substrate p62 was notably reduced in DATS group compared to NC group, suggesting that DATS could induce autophagy in OS cells. Furthermore, OS cells were transfected with a pEGFP-C3-MAP1LC3B reporter, the autophagosomes was examined by confocal imaging. The results revealed that the content of autophagy vesicles in DATS group was higher than that in NC group, where autophagosomes were indicated by green signal. (Fig. 2B). This founding was consistent with the alternation of Western blot analysis. Additionally, flow cytometric assay was performed to investigate the effect of DATS on cell apoptosis. As we can see in Fig. 2C, the apoptosis rate in the blank group was only 1.46%, whereas it increased to 16.20% after DATS intervention.

### 2.3 DATS induced autophagy via EGFR/PI3K/AKT/mTOR pathway

**Fig. 3:**
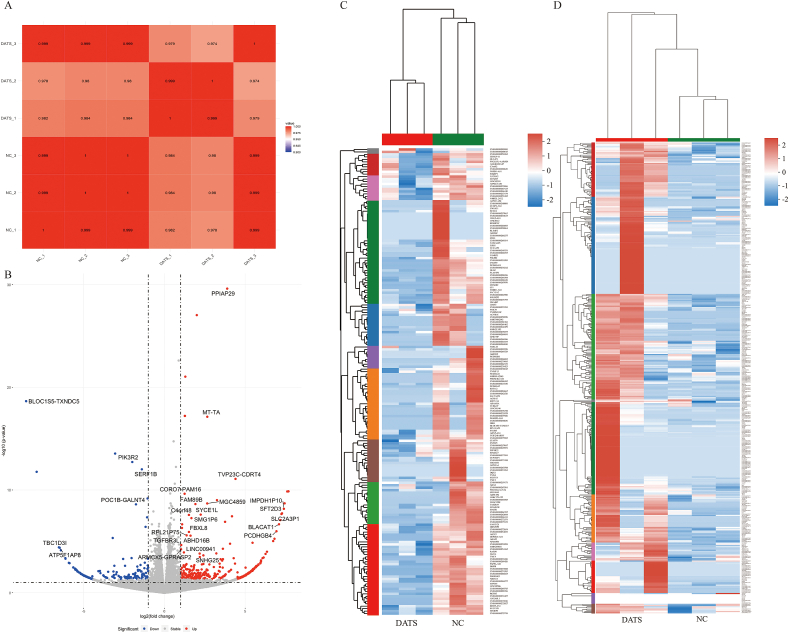
The overview of RNA-seq. (A) Pearson correlation map between each sample of RNA-seq. (B) Volcano map of differential expressed genes after DATS treatment. (C) Heatmap of up-regulating expressed genes after DATS treatment. (D) Heatmap of down-regulating expressed genes after DATS treatment.

**Fig. 4:**
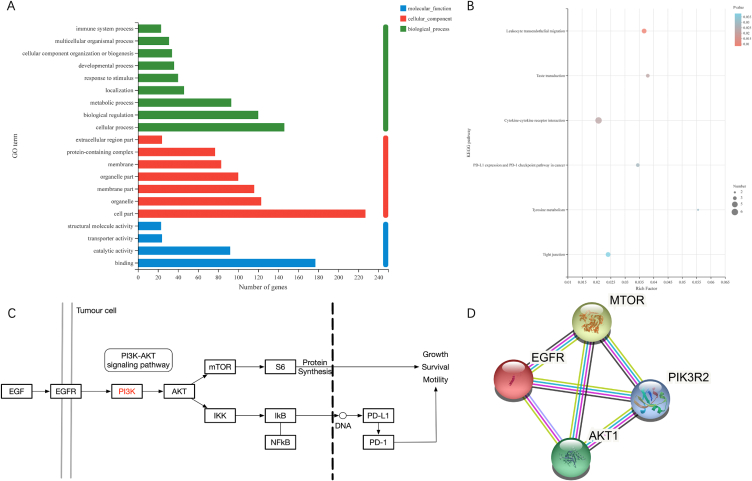
Bioinformatic analysis of RNA-seq data. (A) The histogram image of top 20 altered GO terms after DATS treatment in GO enrichment results. (B) The scatterplot image of KEGG pathways after DATS treatment in KEGG enrichment results. (C) The EGFR/PI3K/AKT/mTOR signaling pathway in PD-L1 and PD-1 pathway. (D) PPI network for EGFR and PI3K/AKT/mTOR signaling pathway.

To further investigate the underlying molecular mechanism of DATS on OS cells, the RNA sequencing (RNA-seq) method was used to analysis the differentially expressed genes in 143B cells treated with 100 μM DATS and NC. The RNAseq dataset described 392 upregulated genes and 189 downregulated genes in 143B cells upon DATS treatment, indicating a great alterations in gene expression between DATS-treated cells and untreated cells (Fig. 3 A, C, D). Notably, PIK3R2 exhibited prominent expression in the volcano map (Fig. 3B). Then, GO and KEGG analyses was performed to elucidate the signal transduction pathways potentially influenced by DATS treatment. The enrichment results identified a total of 225 rich GO terms as *P* < 0.05. In Figs. 4A and 20 most diverse GO terms were enriched in 143B cells treated with DATS. Among them, the expression of PD-L1 and PD-1 checkpoints was activated for tumor inhibition. Based on our findings, it is indicated that DATS may primarily modulate the proliferation of OS cells mainly by promoting the PD-L1 and PD-1 checkpoint pathways. Next, a KEGG enrichment analysis was conducted to identify the altered signal pathways upon DATS treatment. Interestingly, PD-1 signal pathway also was enriched when cells were treated with DATS (Fig. 4B). All these results collectively suggested that DATS was a potential regulator of PIK3R2 and may be involved in PD-1 and PD-L1 signal pathway.

The significant correlation between PI3K/AKT signaling and the inhibition of DATS in OS cells was demonstrated in Fig. 4C. Previous reports had highlighted the regulatory influence of EGFR on the PI3K/AKT/mTOR signal pathway [28]. Our KEGG pathway enrichment analysis mapped that EGFR is an upstream protein of the PI3K/AKT/mTOR signal pathway (Fig. 4C). To investigate the interaction between EGFR and PI3K/AKT/mTOR pathway, we constructed a protein-protein interaction (PPI) network. The results of the PPI analysis predict that EGFR could bind to PIK3R2, AKT1 and mTOR (Fig. 4D). Based on these findings, we selected the EGFR/PI3K/AKT/mTOR as the candidate signaling pathway influenced by DATS in OS cells.

**Fig. 5:**
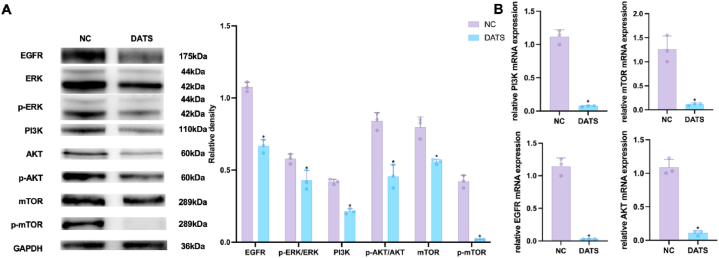
DATS regulated EGFR/AKT/mTOR signaling pathway. (A) Western blot was employed to measure the relative expressions of EGFR, *p*-ERK/ERK, PI3K, *p*-AKT/AKT, mTOR and *p*-mTOR in OS cells. (B) qPCR was used to measure the expression of PI3K, mTOR, EGFR and AKT in OS cells. *p < 0.05 (with NC). Data are represented as means ± SD; n = 3 per group.

Subsequently, we assessed the expression of phosphorylated mTOR, along with its upstream proteins EGFR, Extracellular signal regulated kinases 1 and 2 (ERK1/2), Phosphorylated forms of ERK1/2 (*p*-ERK1/2), PI3K, AKT and *p*-AKT to investigate whether DATS induced autophagy through EGFR/PI3K/AKT/mTOR pathway. In Fig. 5A, the expression of EGFR, ERK, *p*-ERK, PI3K, AKT, *p*-AKT, mTOR and *p*-mTOR was downregulated in the DATS-treated group compared to the NC group. To validate these findings, we found that qRT-PCR results was consistent with the results of Western blot analysis. DATS treatment inhibited the mRNA levels of EGFR, PI3K, AKT, and mTOR in comparison to the control group (Fig. 5B). These findings confirmed the deactivation of the EGFR/PI3K/AKT/mTOR pathway in OS cells treated with DATS.

### 2.4 DATS inhibited the proliferation, migration, invasion and EMT, promotes apoptosis of OS cells by inducing autophagy via EGFR/PI3K/AKT/mTOR signaling

**Fig. 6:**
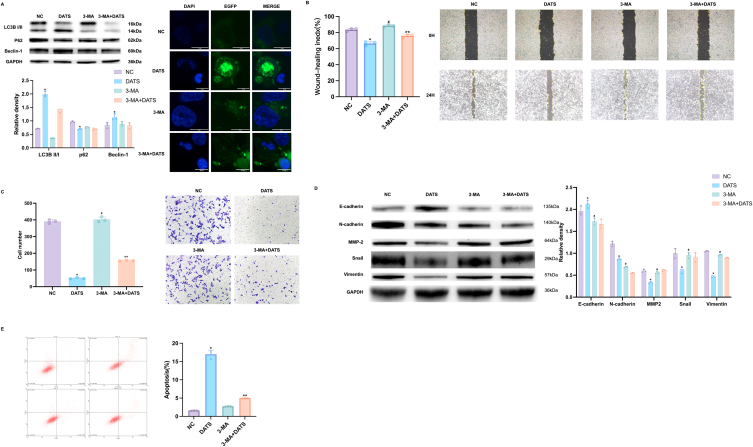
Autophagy can be reversed by 3-MA. (A) Western blot was employed to measure the expression of LC3 I/II, p62 and Beclin-1, OS cells were transfected with pEGFP-C3-MAP1LC3B reporter and analyzed with confocal microscopy images. Scale bar: 10 μm. (B–C) Wound healing and transwell were used to assess the potential of DATS and 3-MA on migrative and invasive abilities. Scale bar: 1 mm for B and 500 μm for C. (D) The expression levels of E-cad, N-cad, MMP-2, Snail and Vimentin in OS cells was measured by Western blot. (E) Flow cytometric assays showed the apoptosis rate of OS treated with DATS and 3-MA. *p < 0.05 (with NC). #*p* < 0.05 (with DATS). **p < 0.05 (with NC and DATS). Data are shown as means ± SD; n = 3 per group.

To elucidate the role of autophagy in modulating the anti-tumor activity of DATS in OS cells, we employed 3-methyladenine (3-MA), a known PI3K and autophagy inhibitor, to impede autophagic processes and assessed the consequent alterations in DATS-induced autophagy markers. As previous results, DATS treatment led to a significant increase in the conversion rate from LC3B–I to LC3B-II, as well as upregulation of Beclin-1, but a reduce of the expression of p62. The treatment of 3-MA can partially rescue the changes in LC3B-II and Beclin-1 induced by DATS. Moreover, the DATS+3-MA group exhibited a decrease in the number of autophagosomes, indicating that 3-MA successfully suppressed the autophagy which induced by DATS (Fig. 6A).

Next, we further estimated whether autophagy inhibitor 3-MA could reverse the effect of DATS on OS cells growth, migration, invasion and EMT. As shown in Fig. 6B and C, treatment with DATS significantly reduced the migration and invasion abilities of OS cells. However, pre-treatment with 3-MA markedly reversed the inhibition effects of DATS, restoring the migration and invasion capabilities of the cells. In addition, the expression of EMT-related proteins was tested by Western blot. In Fig. 6D, we observed that DATS treatment significantly increased the expression of E-cad, inhibited the levels of N-cad, Snail, MMP2 and Vimentin. Pre-treatment with 3-MA counteracted the effects of DATS on these EMT-related proteins. Furthermore, we performed flow cytometry to assess the apoptotic ability of OS cells after DATS or combination with 3-MA. The results depicted in Fig. 6E revealed that treatment with DATS significantly increased the apoptotic ratio by 17.36%, compared to NC group (with an apoptotic ratio of 1.54%). The 3-MA incubation partly reduced the percentage of apoptotic OS cells to 5.12% which was induced by DATS treatment. These findings suggested that DATS can effectively inhibit autophagy-associated migration, invasion, EMT and promote OS cells apoptosis.

**Fig. 7:**
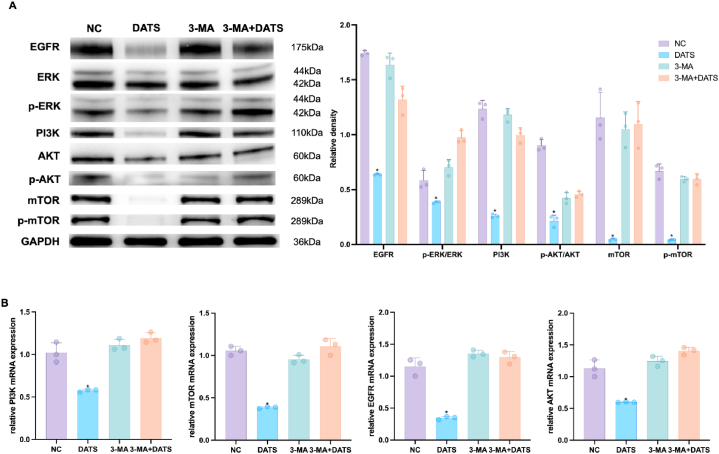
DATS inactivated EGFR/PI3K/AKT/mTOR pathway. (A) Western blot was used to detect the EGFR, ERK, *p*-ERK, PI3K, AKT, *p*-AKT, mTOR and *p*-mTOR expression. (B) qRT-PCR was used to examine the expression of PI3K, mTOR, EGFR and AKT in OS cells after DATS and/or 3-MA treatment. *p < 0.05 (with NC). Data are shown as means ± SD; n = 3 per group.

In our previous results, PI3K was closely related to the effect of DATS. We next explored the underlying mechanism of DATS-induced autophagy by checking the activity of EGFR/PI3K/AKT/mTOR pathway. As shown in Fig. 7A and B, the results from Western blotting and qRT-PCR demonstrated that DATS treatment decreased the expression levels of EGFR, *p*-ERK/ERK, *p*-AKT/AKT and *p*-mTOR/mTOR. Importantly, pre-treatment with 3-MA reversed these effects, indicating that DATS influenced the activation of EGFR/PI3K/AKT/mTOR pathway.

Correspondingly, treatment with 3-MA abolished the autophagy-inducing effect of DATS, including cell migration, invasion, EMT and apoptosis by upregulating EGFR/PI3K/AKT/mTOR signaling pathway.

## 3 Discussion

Despite the current treatments such as chemotherapy and surgery [29] has made great progress, OS remains challenging to treat due to factors like chemotherapy drug resistance, post-operative recurrence, and tumor metastasis [[30], [31], [32]]. Therefore, there is a need to explore novel treatment approches to improve the outcomes for OS patients.

Traditional Chinese Medicine has been shown to fight against bone tumors by targeting various genes and pathways. For example, studies by Ma et al. [33] demonstrated that Cinobufagin inhibited bone tumor progression and reduced doxorubicin resistance through FOXO1-mediated FCGBP transcription. Yu et al. [34] showed that Salvianol promoted apoptosis in U2 cells by activating p53. Additionally, Zhang and his team [35] reported that Baicalin inhibited osteosarcoma progression in vivo and in vitro by downregulating the Wnt/β-catenin pathway.

As the main active component found in garlic, DATS has exhibited inhibitory effects on the proliferation and induction of apoptosis in breast cancer cells [36]. It has also been shown to suppress bladder cancer by inhibiting migration, EMT, and inducing autophagy [37]. Evidently, some progress has been made in the study of DATS in the treatment of bone tumors. For instance, Xie et al. [24] found that DATS inhibited the growth of Saos-2 OS cells and upregulated calreticulin expression. This team also discovered that allicin inhibited OS growth through oxidative stress and the inhibition of the lncRNA MALAT1-miR-376a-Wnt/β-Catenin signaling pathway [24]. Moreover, DATS has been shown to reverse P-glycoprotein-mediated multidrug resistance in OS [38]. However, it is not yet known whether DATS can inhibit OS progression by regulating the EGFR pathway and its downstream signaling. In this study, we have identified, for the first time, that DATS can inhibit migration, invasion and EMT in 143B OS cells through the EGFR/PI3K/AKT/mTOR signaling pathway.

While numerous investigations, such as the one by He et al. [26], have significantly advanced our knowledge on osteosarcoma treatments by elucidating apoptosis-centric mechanisms, autophagy, despite its crucial role in cellular homeostasis and potential therapeutic relevance, has not been as thoroughly interrogated. Our work diverges notably from this trend, prioritizing autophagy within the 143B osteosarcoma cell line as a central theme of inquiry. This focus has permitted us to uncover the modulatory effects of DATS on autophagic processes, positioning our findings as a crucial addition to the body of knowledge. Such pioneering insights open new therapeutic perspectives and underscore the importance of autophagy as a potential target in the development of innovative osteosarcoma treatments.

Autophagy is a normal physiological process involved in maintaining cellular homeostasis and preventing various diseases [39,40]. Overactivation of autophagy has been shown to induce tumor cell death [41,42]. Beclin-1 and LC3II are involved in the formation of autophagosome [43], while p62 expression reflects the degradation of autophagosomes following their fusion with lysosomes [44]. Previous studies have demonstrated the potential of autophagy inducers in treating malignancies [45]. More significantly, several experiments have confirmed that the activation of autophagy inhibits the growth of OS and induces apoptosis [46]. Thus, in this study, we investigated whether autophagy was correlated to the inhibitory effects of DATS on OS cells. To evaluate cellular autophagy, we first assessed the expression of key autophagic markers Beclin-1, LC3II and p62. For the recuse experiments, we employed 3-MA, a well-known autophagy inhibitor to block autophagy [40,47,48]. Our study found that DATS can induce autophagy by modulating the expression of autophagy-related proteins. Furthermore, this induction of autophagy promoted apoptosis in OS cells, ultimately leading to the suppression of OS progression.

Epidermal growth factor (EGF) was first discovered by Dr. Cohen in 1962, and subsequent research revealed that EGF activates its receptor, EGFR, which plays important roles in cell differentiation, apoptosis, proliferation, and migration [49,50]. This enables the targeting of EGFR for the treatment of proliferative diseases [51]. In addition, EGFR expression is significantly elevated and dysregulated in a variety of neoplastic diseases [52,53]. Targeting EGFR has been shown to promise in the treatment of many cancers, such as breast, lung cancer and glioblastoma, through PI3K-AKT and EGFR-RAS-RAF [[54], [55], [56], [57]]. In the context of OS, EGFR has been found to be closely associated with its development. For instance, by using a bioinformatics approach, Zhao et al. [58] found that epi-EGFR, a central gene, was significantly linked with the prognosis of OS patients and was shown to act as an independent prognostic factor. Additionly, EGFR has been identified as an important gene which is associated with autophagy in OS, functioning through the PI3K and MAPK signaling pathways [59]. MiR-491-5p has been reported to suppress tumor growth in osteosarcoma by reducing EGFR expression [60]. Linder et al. [61] found that deletion or inhibition of EGFR directly inhibited the proliferation of osteosarcoma cells by constructing autologous c-Fos-dependent OS mouse and human biopsy samples for preclinical studies. Moreover, EGFR inhibitors have exhibited significant anti-tumor activity against OS [12]. The EGFR/PI3K/AKT/mTOR pathway plays critical roles in various cellular processes, including cell growth, apoptosis, and metabolism in various tumors. In our study, we observed that DATS treatment significantly reduced the expression of EGFR protein and mRNA in 143B cells, downregulated the downstream PI3K/AKT/mTOR pathway. Consequently, cell proliferation was inhibited, and apoptosis was promoted by DATS in OS cells.

Furthermore, a study by Zhao et al. [62] demonstrated that STF cDNA weakened the PI3K/AKT/mTOR axis, enhancing autophagy could slow down the onset and metastasis of OS. Our study found that DATS significantly inhibited EGFR expression and induced autophagy, which played a vital role in apoptosis and migration. This inhibitory effect of DATS can be reversed by adding 3-MA, indicating the reactivation of the EGFR/PI3K/AKT/mTOR signaling pathway partly increased migratory and invasiveness, but decreased expression of autophagy-related markers. Therefore, DATS could induce OS cells autophagy, promote cell apoptosis, and inhibit cell growth by suppressing EGFR/PI3K/AKT/mTOR pathway.

It is essential to recognize the limitations inherent in our investigation, which focused narrowly on the 143B osteosarcoma cell line. This particular cell line does not encapsulate the extensive heterogeneity characteristic of osteosarcoma subtypes in their entirety. Although the selected 143B cell line is a prevalent model in the study of high-grade osteosarcoma, it would be imprudent to assume that the implications of our findings are universally applicable to all osteosarcoma cell variants. Given the notable genetic and phenotypic diversity of osteosarcoma and the corresponding variability in therapeutic responses among different cell lines and clinical presentations, it becomes clear that further research is imperative. Subsequent studies should endeavor to include a diverse array of osteosarcoma cell lines, each with unique genetic profiles, to more comprehensively assess the translational potential and therapeutic efficacy of DATS throughout various manifestations of the disease. By broadening the scope of investigation to incorporate these additional cell models, we anticipate that the resultant data will significantly enhance our understanding of DATS's clinical applicability and effectiveness as an intervention for osteosarcoma.

In summary, this study confirmed that EGFR is an important target of DATS in the treatment of OS. DATS induced autophagy, suppressed cell migration and promoted apoptosis by inhibiting EGFR and its downstream signaling pathway. This inhibitory effect was significantly attenuated by the addition of 3-MA. Thus, this study suggested that DATS inhibited the growth of 143B cells by inactivating the EGFR/PI3K/AKT/mTOR axis, thereby promoting autophagy. These results highlight the potential of DATS as an important therapeutic agent for the treatment of OS.

## 4 Materials and methods

### 4.1 Sample preparation

The molecular weight of DATS (#D3202, LKT Labs, USA) is 178.34 and the purity is 99.2%. 50 μl DATS mother liquor was weighed to calculate the concentration of the mother liquor, which is 6.28 mol/L. DMSO(#D2650, Sigma, Germany) was used to dilute the concentration of DATS to 400 μM in 2 ml centrifuge tubes (Corning, USA). They were stored in a refrigerator (Siemens AG, Germany) at −20 °C away from light.

### 4.2 Cell culture

Human osteosarcoma 143B cells were bought from the ATCC (USA). The OS cells were cultured in complete medium 90% Dulbecco's modified Eagle's medium (#SH30022.01, Hyclone, USA) + 10% Fetal bovine serum (#SH30406.06, Hyclone, USA) + 1% streptomycin and penicillin (#15140122, Gibco, USA) in a temperature incubator (Thermo Fisher, USA).

### 4.3 Cell proliferation assay

Osteosarcoma cells were inoculated in a plate (96-wells) (Corning, USA) with 5000 cells per well and cultured in 37 °C cell incubator overnight. Then the cells were treated with DATS for 24 and 48 h. After that, add 10 μl of cell counting kit8 (CCK8) (#CK04, Dojindo, Japan) reagent to each well and incubated at cell incubator for 2 h. Measure the absorbance at 450 nm of each hole using a microplate reader (BioTek, USA).

### 4.4 Microscopy examination of the expression of EGFP-LC3

The 143B cells (1 × 10^5^) were seeded into a 6-well plate (Corning, USA). The plasmid pEGFP-C3-MAP1LC3B (Biogot Biotechnology, China) was transiently introduced into cells following the manufacturer's instructions with Lipofectamine™ 3000 (Thermo Fisher, USA), then observed under a confocal microscope (OLYMPUS, Japan).

### 4.5 Cell apoptosis detection

The cells were placed 2000 cells per well inoculated in a plate (24-wells) (Corning, USA), and growed in different treatments. Use FITC Annexin V Apoptosis Detection Kit I (#556547, BD Biosciences, USA), and analyze by CytoFLEX S flow cytometer (Beckman Coulter, USA) following by the manufacturer's instructions.

### 4.6 Wound healing assay

The osteosarcoma cells were inoculated at 10,000 cells into 6-well plate. Scratched with a 10 μl pipette tip. Cells were photographed under a microscope after wounding at 0, 24 and 48 h.

### 4.7 Cell invasion assay

Cell invasion experiments were performed using transwell chambers (Corning, USA), 10,000 cells were added to each upper chamber, and with DATS in the lower chamber. 24 h later, crystal violet staining (Biyuntian, China) was used to observe and take pictures under a microscope (OLYMPUS, Japan).

### 4.8 Quantification real-time PCR

Total RNA was extracted and reverse transcribed into cDNA and PCR analysized by the ReverTra Ace qPCR RT Kit (TOYOBO, Japan), SYBR Green Real-time PCR Master Mix (TOYOBO, Japan) and QuantStudio Dx (Invitrogen, Thermo Fisher). GAPDH was used as a reference control and the expression was processed using the 2^−ΔΔCt^ method.

The following specific primers used for qPCR was listed as shown in Table 1.

**Table 1:** Primers were used for qPCR.

Gene name	Primers (5′–3′)
PI3K	Forward: CTGCCTGCGACAGATGAGTG
	Reverse: TCCGATTACCAAGTGCTCTTTC
EGFR	Forward: AGGCACGAGTAACAAGCTCAC
	Reverse: ATGAGGACATAACCAGCCACC
AKT	Forward: CTAACTTGAGCCGCAGGAAC
	Reverse: GCTTGCTCAGTTTGCTACCC
mTOR	Forward: CTGATGTCATTTATTGGCACAAA
	Reverse: CAGGGACTCAGAACACAAATGC

### 4.9 Western blot

Total protein was harvested by using RIPA (Biyuntian, China) including protease inhibitors. Blocking with Blocking Buffer (Biyuntian, China), and incubated with primary antibodies Antibodies against E-cadherin (E-cad) (#14472, CST, USA), Extracellular signal regulated kinases 1 and 2 (ERK1/2) (#4695, CST, USA), Snail (#3879, CST, USA), Vimentin (#5741, CST, USA), EGFR (#4267, CST, USA), N-cadherin (N-cad) (#3195, CST, USA), Phosphorylated forms of ERK1/2 (*p*-ERK1/2) (#4370, CST, USA), *p*-mTOR (#5536, CST, USA), mTOR (# 2983, CST, USA), *p*-AKT (Ser473) (#4060, CST, USA), AKT (#9272, CST, USA), LC3BI/II(#43566, CST, USA), PI3K(#4249, CST, USA), Matrix metalloproteinase-2 (MMP-2) (#40994, CST, USA), and GAPDH (1:1000) (#5174, CST, USA). Incubated the membranes with appropriate secondary antibodies (#ab205718 & #ab205719, ABcam, USA). Images were captured via film cassette exposure and chemiluminescence imager exposure (Tanon, China). The intensities of proteins were normalized to GAPDH.

### 4.10 RNA-seq assay and bioinformatic analysis

Total RNA were purification, library construction, and sequencing according to the manufacturer's instructions (Illumina, CA) in Majorbio Bio-pharm Biotechnology (China). The library screened the target fragment of 300 bp and then amplified 15 cycles with Phusion DNA polymerase. After quantification, the DNA was sequenced with Illumina NovaSeq 6000 sequencer (reading length was 2 × 150 bp). Differential expression analysis was |log2 (fold-change)| ≥ 1, and P-adjust ≤ 0.05 by using R package edgeR. Gene Ontology (GO) was performed with the R package GOplot, and he R package clusterProfiler was used to enrichment analysis Kyoto Encyclopedia of Genes and Genomes (KEGG) pathway. The enrichment analysis was considered to be of statistical significance when the p-value < 0.05. Likewise, protein–protein interaction (PPI) was using STRING to construct related networks of signaling pathway proteins.

### 4.11 Statistical analysis

All experiments were tested repeated at least three times. Data were expressed as means ± standard deviation (means ± SD). The softwares of GraphPad Prism 9 and Statistical Product and Service Solutions were used for the data analyses. The differences between different groups were use two-way ANOVA and Tukey multiple comparisons test. It was considered to be statistically significant when *P* < 0.05.

## 5 Conclusion

In conclusion, our results demonstrated that DATS induces autophagy by inhibiting EGFR/PI3K/AKT/mTOR signal pathway, thus inhibiting OS migration, invasion, and EMT. Our study provided strong evidence for supporting DATS as a potential autophagy inducer that may be an effective anti-cancer agent for OS therapy.

## Funding

This work was funded by 10.13039/501100004608Natural Science Foundation of Jiangsu Province (2022 BK20220464, BK20221420), Jiangsu Provincial Traditional Chinese Medicine Science and 10.13039/100006180Technology Development Plan Project (2020 ZD202008), Science and technology projects in Jiangsu Province (2019 BE2019765), Postgraduate Research & Practice Innovation Program of Jiangsu Province (2022 SJCX22_0854) and Natural Science Foundation of 10.13039/501100007956Nanjing University of Chinese Medicine (2022 XZR2021028).

## Data availability statement

The RNA sequencing data that support the findings of this study are accessible in the NCBI Sequence Read Archive (SRA) and can be found at https://www.ncbi.nlm.nih.gov/sra. The specific accession number for this dataset is PRJNA1048917. All other data relevant to this study are included within this publication.

## CRediT authorship contribution statement

**Xiyu Liu:** Writing – review & editing, Software, Investigation, Formal analysis, Conceptualization. **Nan Wang:** Writing – original draft. **Zhiwei He:** Investigation. **Chen Chen:** Investigation. **Jun Ma:** Investigation. **Xin Liu:** Writing – review & editing. **Shan Deng:** Writing – review & editing, Supervision, Resources, Project administration, Investigation. **Lin Xie:** Writing – review & editing, Supervision.

## Declaration of competing interest

The authors declare that they have no known competing financial interests or personal relationships that could have appeared to influence the work reported in this paper.
